# The mechanistic link between health and gut microbiota diversity

**DOI:** 10.1038/s41598-018-20141-6

**Published:** 2018-02-01

**Authors:** Olaf F. A. Larsen, Eric Claassen

**Affiliations:** Vrije Universiteit Amsterdam, Athena Institute, De Boelelaan 1085, 1081 HV Amsterdam, The Netherlands

## Abstract

Although numerous reports link a decreased diversity of the gut microbiota to a declined health status, to date no mechanistic motivation for this exists. Here, we show by applying first principles basic graph theory on small networks that higher diversity within such a network indeed leads to more efficient systems and redundancy. Our results quantitatively support earlier hypothetical considerations on gut microbiota richness with respect to these parameters. Our simulations show that higher species diversity leads to higher resilience within small microbiological ecosystems, like being present in the gut microbiota. This notion should provide an ingredient when developing new interventional strategies within the domain of microbiota management.

## Introduction

The gut microbiota has been a topic of immense interest over the last years, as its composition and diversity seem to be intimately linked to health and disease^1^. Modulation of the microbiota by external factors like, e.g., dietary intervention (prebiotics, probiotics) and faecal transplantation has shown to be a promising therapeutic route to improve numerous health conditions, ranging from anxiety disorders^2^ to recurrent *Clostridium difficile* infections^3^. The gut microbiota harbours a huge number of microorganisms, being approximately equal to the number of human cells^4^. Hence, it comprises an inherently complex network of microbe-microbe and microbe-host interactions^5^. Recent insights suggest that the human gut microbiota contains microbial guilds^6^. Microbial guilds are relatively small groups of microbial species that interact with each other or share a metabolic process^7^. As such, they provide an interesting link between the understanding of processes that take place at single cell level and those that take place at large scale microbial communities. Cross-feeding or syntrophic systems provide an example of small microbial systems, starting at systems comprising only two members^8^. Here, we simulate such microbial systems using elementary network theory, to explore the landscape of possibilities considering connections within the network. The usage of network theory has earlier proven to be a helpful tool to systematically analyse microbial interactions, to understand the underlying physiological mechanisms exerted by the gut microbiota^9^. Our novel results indicate that a higher diversity of the microbial composition leads to a more efficient system, providing a mechanistic base of the general notion that a more diverse microbiota is associated with improved health status.

## Methods and Results

**Figure 1:**
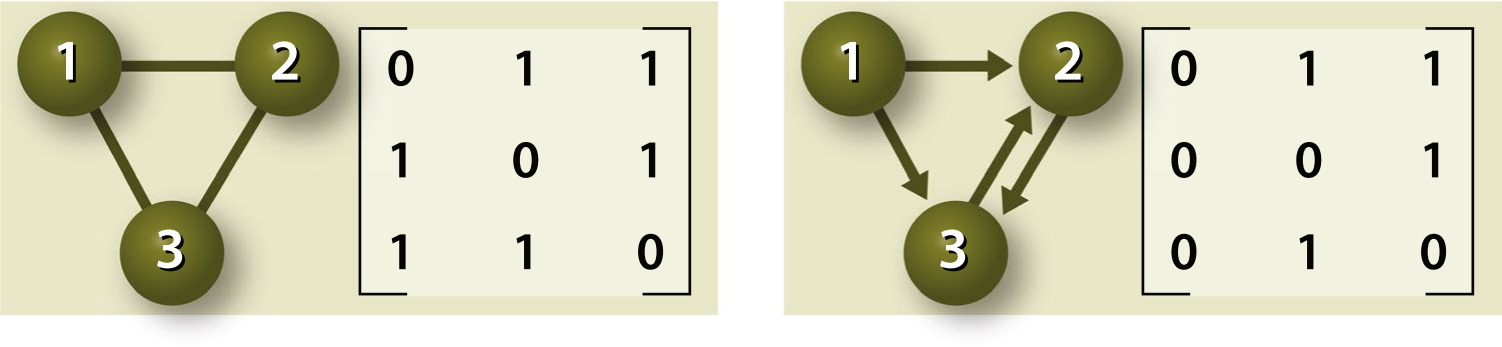
Schematic representations of two systems comprising three microbial species. The circles (“nodes”) represent the three species, whereas the lines (“edges”) represent the signalling connections between the species. In the left panel, the signals are undirected. As such, the presence or absence of an edge between two species simply represents communication or no communication at all between the respective species. In the right panel, the edges are directed. In this example, species 1 can exert a signal to species 2 and 3. Species 2, however, cannot exert a signal to species 1, but can exert a signal to species 3, and species 3 can again exert a signal to species 2. For each graphical representation, the respective adjacency matrix is given as well, with both row- and column-numbers representing nodes 1, 2 and 3, respectively. Each matrix element represents either a connection (“1”) or no connection (“0”) between the species. As an example, for the left adjacency matrix, elements (1, 2) and (2, 1) indicate a connection between species 1 and 2. The right adjacency matrix indicates that there is a signal going from species 1 to species 2, element (1, 2), but there is no signalling “back” possible from species 2 to species 1, element (2, 1). In our simulations, no connection of a species with itself is possible, hence the diagonal elements are all zero. In the case of undirected edges, the matrix is obviously symmetric (left example).

A small system comprising, for example, three microbial species can be viewed by a graph-theoretical representation as depicted in Fig. 1.

**Figure 2:**
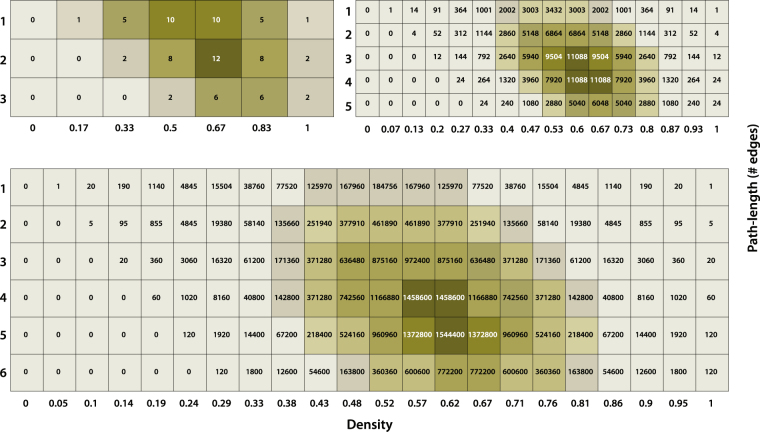
Heatmaps displaying the landscapes of paths with respect to density and path-length. The matrices represent the number of paths possible for configurations of respectively 4 nodes (top left), 6 nodes (top right), and 7 nodes (bottom), when going from node 1 to node n, as function of density (x-axis) and path-length (y-axis). For 7 nodes, the highest numbers of paths possible are centred around a density of 0.62 with path-lengths around 4 to 5.

Although interactions between microbial species are known to be directed^10^, we will make use of undirected graphs as a first estimation. Only simple graphs were being considered in the simulations performed, meaning that they are unweighted, undirected, and do not contain loops or multiple edges. First, we calculated all adjacency matrices possible for systems comprising *n* nodes, with *n* ranging from 2 to 7. As the number of adjacency matrices is given by 2^x^, with $x={\sum }_{i=1}^{n-1}(n-i)$, the number is going up quickly resulting in already 2.097.152 adjacency matrices for only 7 nodes. For each adjacency matrix, the density was calculated as well. The density *D* of an adjacency matrix is given by: $D=\frac{E}{m}$, with *E* being the number of edges present for the specific configuration investigated, and *m* the maximum number of edges possible for the number of nodes given. In a typical metabolic microbial network (guild), a starting species produces a signal (metabolite) for a next species, and so on, to finally reach the final species that produces the “end-product” of the guild. Hence, to explore the associated signalling-landscape between the species, we calculated for each adjacency matrix (a specific microbial signalling configuration) all pathways possible starting from node 1 (“starting node”) and going to node *n* (“target node”), providing all signalling routes going from species 1 to species *n*. We calculated simple paths only, hence every node can only be visited once maximally. Subsequently, we plotted the number of pathways possible as a function of both the path-length (number of edges travelled when going from node 1 to node *n*), and the density. The results for 4,6 and 7 nodes, respectively, are given in Fig. 2.

**Figure 3:**
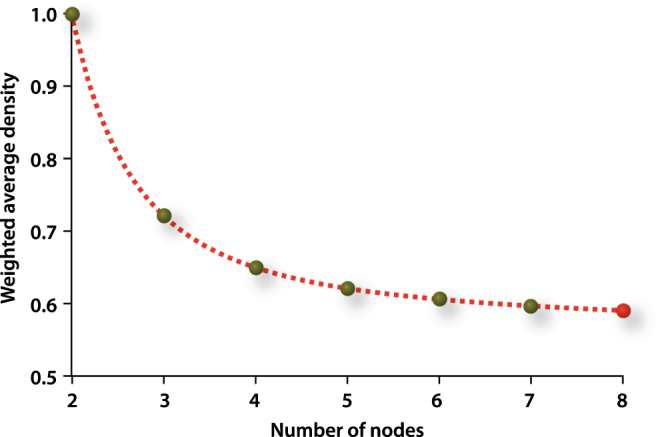
Weighted average density as function of number of nodes. Red dot: not calculated, but obtained from extrapolation using a fit of the calculated data-points. The fit (dotted line, r > 0.999) is added as a guide to the eye.

Finally, we calculated for each heatmap as depicted in Fig. 2 (with *n* ranging from 2 to 7) the average density, weighted by the number of paths. The result is depicted in Fig. 3.

## Discussion

Clearly, the weighted average density is going down with increasing number of nodes. Hence, the average number of connections that a species needs to have with other species to reach the maximum paths possible to go from start to target node (some kind of cross-feeding/syntrophic functionality), is going down upon increasing the number of nodes. This implies a more “relaxed” state of the species: less interaction with other species is needed when the system is more diverse, to reach the final target node. Hence, as relatively less interactions are needed, relatively less energy is consumed, resulting in a more efficient system. A higher efficiency upon higher species diversity is for example observed in mercury-reducing biofilms^11^. Our simulations quantitatively substantiate the notion that the functionality accommodated by small microbial guilds gets more efficient upon introduction of more species. This mechanism is analogous to the reduction of functions within microbial communities upon symbiosis, as reported earlier^12^. Genome reduction can be observed following microbial symbiosis which inherently leads to a more limited repertoire of functions, and as such specialization towards a specific process/metabolic functionality of the new community. Such a new community can also be envisioned within the context of a holobiome, in which the microbiota of an organism “acquires” the microbiota of a symbiont in order to evolve towards a next level organism^13^.

Another interesting feature is the levelling-off of the weighted average density. This supports earlier hypotheses that higher diversity leads to more stability of the microbial system which is associated to redundancy^14^. In a redundant system, removal of species does not automatically result in a loss of functionality of the system. In our picture: removal of species does not pose an extra burden on the remaining species to interact more in order to reach the target, while maintaining the highest signal strength that is possible for the total number of nodes remaining. This starts to be the case from ~6 nodes according to our simulations. It should be stressed that our simulations for these types of small microbial guilds show redundancy in the sense of efficiency of signal transduction. This is different from redundancy in the sense of functional overlap between species. The type of cooperation we anticipate seems to be in line with evolutionary game theory, which earlier showed synergistic effects following the interaction between microbial populations^15^.

**Figure 4:**
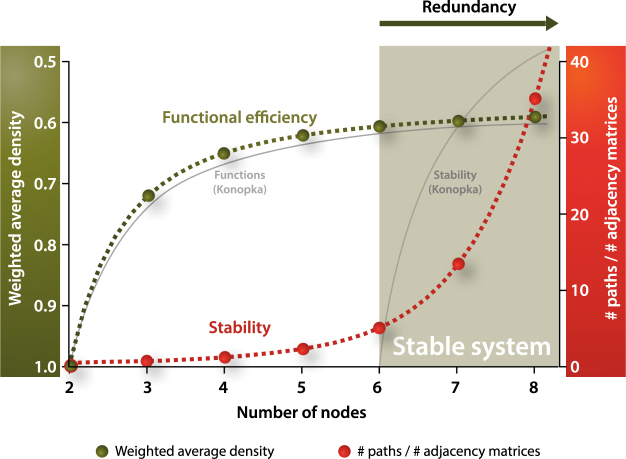
Graphical summary of our findings (adapted from Konopka^14^). Green: weighted average density reflecting functional efficiency. Red: number of paths divided by the number of adjacency matrices, reflecting stability. Grey: trends as proposed earlier by Konopka^14^.

A graphical representation summarizing our findings can be viewed in Fig. 4. This picture is a quantitative substantiation of a conceptual model proposed earlier^14^. In this conceptual model, it was speculated that increasing species richness leads to higher functionality. In our view, for small microbial guilds, increasing species richness leads to higher efficiency of the functionality accommodated by the microbial guild. In the conceptual model, it was hypothesized that stability of the system starts when the functionality starts to level off. Our simulations quantitatively support this view. When plotting the number of paths per adjacency matrix as a function of number of nodes, reflecting the stability of the system, we indeed observe a rapid rise again starting at ~6 nodes.

It might be possible that the behaviour of the weighted average density at higher numbers of nodes (>7 nodes) is different and does not follow the asymptotic trend as depicted in this article. Also, the stability might follow a different trend at higher numbers of nodes. However, we hypothesize that this possible change may be caused by the flipping of an initial ecological state towards an alternative state, which has been proposed before^16^. Such “tipping” from one state to another has recently also been described for the ecosystem of the human intestinal tract^17^.

The end-signal of small microbial ecosystems as discussed in this paper can ultimately give rise to large scale community effects^7^. However, the specific signalling pathways by a single guild will be limited to systems comprising only a small number of species, and will be spatially confined. Experimental evidence of this was reported earlier for the peroxidase activity of lactobacilli to inhibit HIV, which could only be accomplished in an *in-vitro* model and not in real life, indeed hinting to a spatially confined mode of action^18^.

In conclusion, we show that we can quantitatively explain essential features of the gut microbiota by applying some basic graph theory. More detailed simulations are needed to predict and explain specific microbiological ecosystems, which seems to be feasible considering the results already obtained with this highly first-principle approach.

## Electronic supplementary material

Supplementary Information
