# Supplementary material for: The mechanistic link between health and gut microbiota diversity
Source: Sci Rep. 2018 Feb 1;8:2183. doi: 10.1038/s41598-018-20141-6 (PMC5794854; doi:10.1038/s41598-018-20141-6)
Supplement: Supplementary file 1 — Supplementary Information [file 41598_2018_20141_MOESM1_ESM.pdf]

The mechanistic link between health and gut microbiota diversity

Olaf F.A. Larsen* & Eric Claassen

Vrije Universiteit Amsterdam, Athena Institute, De Boelelaan 1085, 1081 HV Amsterdam, The Netherlands

* Corresponding author

SUPPLEMENTARY INFORMATION

Simulations. All simulations were performed using R statistical software (version 3.4.0) within an RStudio environment (Version 1.0.143). Simulations were executed on a HP Z640 workstation containing an Intel(R) Xeon(R) CPU E5-2620 v4 @ 2.10GHz, with 64GB RAM. Simulations were partly performed in parallel (multicore).
